# Recombinant human collagen type III microgel: an advanced injectable dermal filler for rejuvenating aging skin

**DOI:** 10.1093/rb/rbaf076

**Published:** 2025-07-28

**Authors:** Yafang Chen, Yihan Zhao, Xinyue Zhang, Yang Sun, Kang Li, Liguo Zhang, Shuang Li, Jie Liang, Kefeng Wang, Yujiang Fan

**Affiliations:** National Engineering Research Center for Biomaterials, Sichuan University, Chengdu 610065, China; College of Biomedical Engineering, Sichuan University, Chengdu 610065, China; National Engineering Research Center for Biomaterials, Sichuan University, Chengdu 610065, China; College of Biomedical Engineering, Sichuan University, Chengdu 610065, China; National Engineering Research Center for Biomaterials, Sichuan University, Chengdu 610065, China; College of Biomedical Engineering, Sichuan University, Chengdu 610065, China; National Engineering Research Center for Biomaterials, Sichuan University, Chengdu 610065, China; College of Biomedical Engineering, Sichuan University, Chengdu 610065, China; Collaborative Innovation Centre of Regenerative Medicine and Medical BioResource Development and Application Co-constructed by the Province and Ministry, Guangxi Medical University, Nanning, Guangxi 530021, China; National Engineering Research Center for Biomaterials, Sichuan University, Chengdu 610065, China; College of Biomedical Engineering, Sichuan University, Chengdu 610065, China; State Key Laboratory for Conservation and Utilization of Subtropical Agro-bioresources, College of Life Science and Technology, Guangxi University, Nanning 530004, China; Harbin Fuerjia Technology Co, Ltd., Harbin 150000, China; Harbin Fuerjia Technology Co, Ltd., Harbin 150000, China; National Engineering Research Center for Biomaterials, Sichuan University, Chengdu 610065, China; College of Biomedical Engineering, Sichuan University, Chengdu 610065, China; Sichuan Testing Center for Biomaterials and Medical Devices, Chengdu 610064, China; National Engineering Research Center for Biomaterials, Sichuan University, Chengdu 610065, China; College of Biomedical Engineering, Sichuan University, Chengdu 610065, China; National Engineering Research Center for Biomaterials, Sichuan University, Chengdu 610065, China; College of Biomedical Engineering, Sichuan University, Chengdu 610065, China

**Keywords:** injectable rhCol III microgel filler, aging skin rejuvenation, inducing collagen regeneration, inducing elastic fiber regeneration

## Abstract

Skin aging, characterized by reduced collagen regeneration, chronic inflammation and heightened skin cancer risk, poses a significant challenge. Collagen-based materials, employed as dermal fillers to smooth wrinkles, have attained extensive utilization. Nevertheless, traditional animal-derived collagen protein primarily presents concerns pertaining to disease risks, potential immunological reactions, and batch instability. Here, we introduced a highly durable 1,4-butanediol diglycidyl ether cross-linked recombinant human collagen type III (rhCol III) microgel as dermal filler for rejuvenating aging skin. The rhCol III microgel exhibited exceptional thermostability, mechanical strength and injectability. Subsequently, we established a UV-photoaging skin animal model and chose rhCol III microgel as a bioactive material for *in vivo* implantation, systematically comparing its biological effect with commercialized collagen I (Col I) derived from porcine skin (pCollagen) and hyaluronic acid through histological observation, immunofluorescence staining, hydroxyproline quantification and analysis of specific gene expression. Outcomes indicated rhCol III microgel prompted augmented production of Col I, collagen III (Col III) and elastic fibers, thereby contributing to the remodeling of the extracellular matrix (ECM). In summary, our investigation contributed to robust biosafety and rejuvenation of UV-induced skin photoaging by rhCol III under a single injection for 6 weeks. Despite the imperative ongoing efforts required for the successful translation from bench to clinic, the discernibly superior safety and efficacy of rhCol III microgel present an innovative methodology in combating skin aging, offering significant promise in medical cosmetology and tissue engineering.

## Graphical abstract

**Graphical Abstract:**
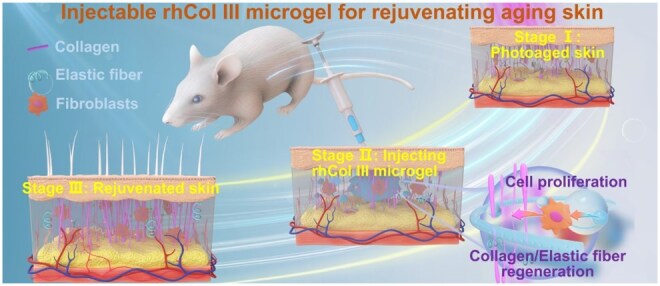

## Introduction

Skin aging, an inevitable physiological process, causes skin to dry, desquamate, thicken, and lose elasticity, followed by the appearance of wrinkles and solar dermatitis, among other skin-related conditions [1, 2]. With the increasing pursuit of rejuvenation, people have attempted many ways to combat skin aging. To protect or repair skin, a number of materials have been used, including antioxidants, retinoids [3], peptides [4] and growth factors [5]. The use of dermal fillers is more effective at smoothing facial contours and has a much longer-lasting effect than topical treatments, with advantages like simple operation, minimal trauma, short recovery time, as well as obvious repair effect [6, 7]. These clinically used injectable bulking agents can be made from synthetic and natural polymers, including silicon, polycaprolactone, calcium hydroxyapatite and hyaluronic acid (HA) [8–10]. Nevertheless, the current materials are not widely used as filler composites due to long-term inflammation, allergic reactions and rapid degradation because of weak mechanical properties [11, 12].

Since collagen is the structural protein of extracellular matrix (ECM), it is widely used as injectable fillers and in tissue engineering studies [13–15]. The skin primarily comprises Col I and Col III, constituting 80–90% and 8–12% of the total content, respectively [16, 17]. Specifically, Col III plays an important role in dermal collagen fibrillogenesis and tissue integrity [18]. Moreover, it has been shown to induce the transcription of growth factors for fibroblasts, including keratinocyte growth factor, vimentin, and transforming growth factor beta (TGF-β) [19, 20]. It has been found that the ratio and content of these two types of collagen vary with age. In normal fetal skin, Col III accounts for about 34–65% of the total collagen, whereas Col I makes up about 80–85% of the total collagen in childhood and adult skin [21]. Meanwhile, the adult dermal matrix loses its capacity for synthesizing Col III, becoming restricted to Col I synthesis. Particularly noteworthy is the observation that the dermal synthesis rate of Col III fails to match the pace of its depletion post the age of 25 [22]. In light of these considerations, the supplementation of exogenous Col III is an important means to impede the progression of skin aging. Currently, predominant methods for Col III preparation encompass acid and alkaline hydrolysis and enzymatic digestion of animal connective tissue, albeit fraught with notable preparation challenges [23, 24]. The inherent drawbacks of these methodologies include elevated costs, the potential for epidemic transmission risks, and the propensity to elicit immune reactions [25, 26]. Furthermore, factors such as religious sensitivities, purification challenges, and batch variability impose significant constraints on the practical utility of animal-derived collagen [27].

Advancements in genetic engineering and synthetic biology have positioned recombinant human collagen that exhibits similar natural human Col III properties as a noteworthy alternative to traditional animal-derived collagen [28, 29]. These characteristics of recombinant human collagen address concerns related to viral hazards and immunogenic responses associated with conventional animal-derived collagen, thereby surmounting challenges linked to clinical efficacy and quality instability arising from variations in animal age and species [30, 31]. This breakthrough marks a pivotal advancement in the realm of collagen-based biomaterials [32]. Wu et al. have demonstrated that the use of an anti-inflammatory rhCol III coating scaffold can lead to accelerated endothelial healing when implanted into injured vascular tissue [33]. Similarly, our research group also confirmed that rhCol III could promote fibroblast proliferation, migration, and collagen secretion, thereby demonstrating promising potential for skin regeneration [34, 35]. Moreover, Wang et al. also demonstrated that rhCol III stimulated collagen synthesis in damaged skin caused by UV irradiation [29]. However, the need for regular replenishment limits its further application.

Chemical cross-linking has garnered significant interest owing to its simplicity relative to alternative approaches and its capacity to enhance cross-linking density, thereby improving enzymatic resistance. Glutaraldehyde (GA) cross-linking of collagen improves the thermal stability and anti-enzymatic activity of collagen. However, GA has a potential risk of cytotoxicity and calcification [36, 37]. While the 1-(3-dimethylaminopropyl)-3-ethylcarbodiimide hydrochloride/N-hydroxysuccinimide (EDC/NHS)-mediated cross-linking strategy enhances the mechanical elasticity of collagen through covalent amide bond formation, it concurrently depletes the carboxylate anion of glutamate, leading to impaired cell spreading, survival and proliferation [38, 39]. Similarly, genipin-cross-linked collagen exhibits enhanced durability; yet, its potential discoloration limits its clinical aesthetic utility [40–42]. These limitations underscore the persistent challenge in developing injectable collagen-based fillers that harmonize biocompatibility and structural durability for clinical translation.

Herein, injectable rhCol III microgel was developed combining genetic engineering, synthetic biology and 1,4-butanediol diglycidyl ether (BDDE) cross-linking technology as dermal filler for rejuvenating aging skin. Preliminary exploration was undertaken to assess the stability and injectability of rhCol III microgel. Then, the research systematically explored the effects of filler materials on key cellular activities, including collagen secretion. Subsequently, the study delved into the post-injection degradation of various filler materials in animal experiments, employing gross observation, hematoxylin and eosin (HE) staining. Furthermore, the research explored the impact of distinct filler materials and injection frequency on collagen regeneration by using Elastica van Gieson (EVG) staining, Sirius red staining, hydroxyproline quantification and comprehensive gene expression analysis in a UV-induced photoaging skin animal model. In brief, the effects of rhCol III microgel, HA, and animal-derived collagen (pCollagen) were compared in terms of filler effect and promotion of collagen regeneration, which had not been studied in previous reports.

## Materials and methods

### Materials

Recombinant human collagen type III (rhCol III, Mw = 55.0 kDa) was kindly purchased from Jiangsu JLand Biotech Co., Ltd. To illustrate the filling effect, commercialized pCollagen (this material comprises cross-linked Col I derived from porcine skin and phosphate-buffered saline) dermal filler and HA (the gel particle suspension is primarily composed of partially modified sodium hyaluronate, sodium chloride, phosphate buffer system, and injectable water, with a labeled concentration of 20 mg/mL) dermal filler were used as controls. α-minimum essential medium (α-MEM), Dulbecco’s modified eagle medium (DMEM) and phosphate-buffered saline were purchased from Hyclone (USA). Fetal bovine serum (FBS) was bought from Thermo Fisher (USA), saline was bought from Sichuan Meida Kangjiale Pharmaceutical Co., Ltd. Polyoxymethylene was bought from Biosharp. Hydroxyproline (BC0250) (HYP) assay kit was bought from Solarbio. Lipopolysaccharide (LPS) was bought from Sigma. RNA extraction and purification kit was bought from TianGen. cDNA reverse transcription kit was bought from Yeasen. Polymerase chain reaction (PCR) reagent kit was bought from Yeasen.

### Preparation of injectable rhCol III microgel

A 3.6 g of recombinant human collagen type III powder (rhCol III^Y^) was fully dissolved in 20 g of physiological saline. The pH of the solution was adjusted to 10 using a 0.5 mol/L sodium hydroxide solution. Then, 0.7128 g of BDDE was added under a 60°C constant temperature water bath for 12 h to allow cross-linking. The resulting gel was cut into approximately 1 cm³ pieces and cleaned by 500 mL of a 50 g/L anhydrous sodium dihydrogen phosphate solution for 2 h. Subsequently, the gel pieces were further cleaned in 500 mL of physiological saline for 8 h. The gel was then homogenized to form cross-linked recombinant human collagen type III (rhCol III microgel).

### Determination of BDDE residue content

Approximately 1.0 g of the sample was weighed accurately. Then, 1 mL of alkaline protease solution was added, and the sample was allowed to dissolve completely. Afterward, 2.0 mL of ethyl acetate was added to the above solution, which was mixed well and centrifuged at 14 000 rpm for 5 min. Finally, transfer the supernatant to an autosampler for analysis by gas chromatography (Agilent 8890). Record the chromatogram and calculate the residual BDDE content X (μg/g) of the sample using the following formula:


X (μg/g)=Cs×Ai×2/(As×Wi),

where Cs is the concentration of the BDDE standard solution in the standard tube (2 μg/mL), Ai is the peak area of BDDE for the rhCol III microgel solution, As is the peak area of BDDE in the standard solution and Wi is the weight of the sample (g).

### Circular dichroism spectroscopy

Circular dichroism (CD) spectra of rhCol III^Y^ and rhCol III (0.5 mg/mL in hyperpure (UP) water) were obtained using Chirascan V100 Spectrometer (Applied Photophysics Ltd., UK) using 0.5-mm quartz cuvettes. The spectrum (180–280 nm) for the UP water was subtracted from each sample spectrum and then smoothed using Savitsky-Golay with a window size of 5. The molar ellipticity [**θ**] was calculated by the following equation:


[θ]=mdeg/(l×c),

where mdeg is the millidegree obtained from CD spectrometer, c is the concentration of sample and l is the optical path length.

### Fourier transform infrared spectrum

About 10 mg of dried rhCol III microgel was examined by attenuated total reflection Fourier transform infrared spectroscopy (Nicolet 6700, Thermo Electron Corporation, USA). The samples were scanned with 16 accumulations and a resolution of 4 cm^−1^ in the wavenumber range of 4000–500 cm^−1^.

### Raman spectroscopy

High-resolution Raman imaging spectrometer (LabRAM Soleil, Horiba France SAS) was used to analyze the molecular structure of the rhCol III microgel under a laser wavelength of 785 nm and a test wave number of 500–2500 cm^−1^. Subsequent data analyses were performed in OriginPro 2021 software.

### Thermogravimetric analysis

The thermal stability of the material was characterized using a thermogravimetric analyzer (TGA 2, Mettler Toledo) over a temperature range of 30–800°C under a heating rate of 10°C/min and an air atmosphere. The mass-temperature data were obtained by further analyzing.

### Differential scanning calorimetry

The thermal behavior of the material was investigated using a differential scanning calorimetry (DSC) 3+ instrument (Mettler Toledo) under an air atmosphere. The sample was first stabilized at 25°C for 5 min and then heated from 25°C to 150°C. The heat flow-temperature data were obtained by further analyses.

### Scanning electron microscope

Dried rhCol III^Y^ and rhCol III microgel were meticulously mounted onto copper stubs utilizing conductive adhesive and subjected to a gold sputter coating. The internal morphological characteristics of the materials were scrutinized employing a scanning electron microscope (SEM) (HITACHI S-800, Japan).

### Transmission electron microscope

The rhCol III microgel (10 μL) was placed on 200 mesh carbon-coated copper grids. The excess solution was removed using filter paper. The sample was stained with 1% (w/v) phosphotungstic acid (10 μL) solution for about 2–3 min and imaged on JEM-2100 Plus (Japan).

### Gel permeation chromatography

The number average molecular weight (Mn) of rhCol III microgel at a concentration of 120 mg/mL was ascertained through gel permeation chromatography (GPC) (Waters 1525, America) at a flow rate of 1 mL/min. The mobile phase consisted of a buffer comprising sodium nitrate (0.20 M) and sodium dihydrogen phosphate (0.01 M). Polyethylene glycol served as the standard molecular weight kit in this analytical procedure.

### Rheological analysis

The rheological properties of rhCol III microgel at a concentration of 120 mg/mL were assessed using a rheometer (MCR302, Anton Paar). Three sets of filler samples underwent oscillatory mode testing to discern the storage modulus (G′) and loss modulus (G″) under varying strain conditions at 37°C. The specific testing parameters involved logarithmically adjusting the strain from 0.1% to 500%, maintaining a constant angular frequency of 10 Hz, with a total of 50 recorded data points. Furthermore, the viscosity alterations of rhCol III microgel were examined in triplicate at 37°C. The specific testing conditions included a data acquisition interval of one point every second, starting from the steady state, resulting in a total of 30 data points. The shear rate was logarithmically varied from 0.1 to 100 Hz.

### Osmotic pressure test

Prepare rhCol III microgel at a concentration of 120 mg/mL. Utilizing a pipette, transfer a defined volume of the specimen into a designated sample tube. Subsequently, insert the sample tube onto the testing probe, with the tip of the probe positioned approximately 2 mm above the bottom of the sample tube. Initiate the measurement process by manually descending the measurement probe into the cryogenic trap, whereby the instrument (OM-819.C, Löser) will commence the testing procedure.

### Injection force

Prepare rhCol III microgel at a concentration of 120 mg/mL. The measurement of injection force was conducted employing an electro-mechanical universal testing machine (Shimadzu Autograph AGS-X, Japan) at a crosshead displacement rate of 30 mm/min, reaching a maximum load of 100 N, utilizing a 1-mL syringe (27 G). Each specimen underwent triplicate measurements for comprehensive analysis.

### Interaction between rhCol III microgel and L929 cells

Aseptically procure rhCol III microgel at a concentration of 120 mg/mL and dilute it with α-MEM culture medium containing 1% penicillin-streptomycin into 5 mg/mL. Subsequently, incubate the mixture on a constant temperature shaker at 37°C for 24 h. The Control group was cultured in α-MEM culture medium consisting of 10% FBS. Each experimental group was represented by three replicate wells. In each well of a 24-well plate, seed 0.5 × 10^4^ cells and incubate them in a 5% CO_2_ container at 37°C.

Fluorescein diacetate (FDA)/propidium iodide (PI) was used to assess cell viability in rhCol III microgel after culture for 3 days. The cells were stained with FDA (5 μg/mL)/PI (5 μg/mL) for 5 min and subsequently examined under a confocal microscope (CLSM, Leica TCSSP 5, Germany). Rhodamine and 4’,6-diamidino-2-phenylindole (DAPI) were employed to visualize the cytoskeleton of cells interacting with rhCol III and the cell area was analyzed by image J. The extract solution of rhCol III microgel was prepared according to GBT16886, and the proliferation of L929 cells in rhCol III microgel was examined through methyl thiazolyl tetrazolium (MTT) assays at 24 and 72 h. Optical absorbance at 570 nm was measured using a Multiscan Spectrum (Varioskan Flash, Thermo Fisher Scientific, USA). All experimental samples were conducted in triplicate.

Cell migration was assessed through cell scratch assay following treatment with rhCol III microgel. Briefly, 1.5 × 10^5^ L929 cells were cultured in medium until reaching 80% confluence, at which point a sterile 200-μl pipette tip was used to create a scratch. Subsequently, the sterile rhCol III microgel and cells were co-cultured under serum starvation, while the Control group received treatment solely with serum-free medium. The cell scratch was photographed using an inverted microscope at 0, 8 and 24 h. The migration rate was quantified utilizing the following formula:


$$\mathrm{Migration}\;\mathrm{rate}\;\left(\%\right)=\left(A_{0}-A_{1}\right)/A_{0}\times 100\%,$$

where A_0_ was the wound area at 0 h and A_1_ was the wound area at a predetermined time point.

Furthermore, perform immunofluorescence staining for collagen I (1:500, ab270993) and collagen III (1:200, ab7778) of the cells cultured on the coverslips.

### Degradation properties of rhCol III microgel in subcutaneous skin in mice

All animal experiments adhered to prevailing guidelines for the ethical treatment of laboratory animals and received approval by the Experimental Animal Ethics Committee of Sichuan University (Approval Number: KS2023471). Six- to 8-week-old SPF Balb/C mice (*N* = 6) were anesthetized with pentobarbital sodium. Subsequently, the dorsal area was shaved, and the back underwent disinfection with iodine solution. A subcutaneous injection of 0.2 mL of rhCol III microgel at a concentration of 120 mg/mL was administered on the right side of the mice, while an equivalent volume of saline was injected on the left side as a control. Macroscopic observations and photographs of the injection site were recorded at 2 and 4 weeks post-material injection. The injection site was dissected later, and the skin underwent fixation, dehydration, embedding, sectioning, followed by HE staining for further analysis.

### Construction of skin photoaging in a rat model

All animal experiments adhered to prevailing guidelines for the ethical treatment of laboratory animals and received approval by the Experimental Animal Ethics Committee of Sichuan University (approval number: KS2023471). A cohort of 134 male SPF-grade sprague-dawley (SD) rats, aged 7 weeks and weighing between 200 and 250 g, was utilized for the study, procured from Chengdu Dashuo Experimental Animal Co., Ltd. A 7 cm × 10 cm area on the backs of SD rats was shaved, and the animals were housed in cages. Subsequently, the skin was exposed to UVA and UVB radiation from a UV light therapy device at a standardized distance of 30 cm. Premature skin aging caused by UVA and UVB was called photoaging [29]. The experimental duration spanned 8 weeks.

The minimum erythema dose (MED), defined as the amount of ultraviolet radiation capable of inducing the slightest visible reddening of the skin, was determined using an ultraviolet irradiation detector. The MED values were identified as UVA 2.24 J/cm^2^, UVB 0.52 J/cm^2^, irradiation time 7.5 min. To establish a UV-induced photoaging skin model, the experimental animals underwent three distinct phases of UV radiation exposure. The first phase, spanning the initial 4 weeks of the animal study, involved subjecting the animals to 1 MED exposure. The second phase consisted of 2 weeks featured an escalated dose of 1.2 MED. The final phase, extending over 2 weeks, exposed the animals to 1.4 MED exposure. This established UV radiation regimen persisted for a cumulative duration of 8 weeks, and the cumulative radiation dose of UVA and UVB was 42.56 J/cm^2^ and 9.88 J/cm^2^, respectively [6].

### Material injection in skin photoaging in a rat model

Partition the dorsal skin region of each rat along the midline of the back into two distinct surgical areas, designated as left and right, resulting in a total of 20 surgical sites. The experimental timeframe spanned a total of 8 weeks. Over the initial 2 weeks, UV radiation induction was administered to initiate the aging process in the rats. In the third week, 0.5-mL material was implanted into the dermis. Continuous intradermal injections were administered to the double injections group at regular intervals. (Single injection group: material injection performed only once; double injections group: samples replenished at 3-week intervals following a single material injection.) Rats were randomly allocated into groups, as outlined in Table 1. UV radiation exposure persisted for 8 weeks. Tissue samples were collected in the second, fourth and sixth weeks following material implantation.

**Table 1.** The main information about the experimental groups and implants

Groups	Implants (left)	Implants (right)	UV radiation	Refill times
Single injection	None	rhCol III	+	0
	Saline	HA	+	0
	pCollagen	pCollagen	+	0
Double injections	None	rhCol III	+	1
	Saline	HA	+	1
	pCollagen	pCollagen	+	1
Senescence	None	None	+	0
Nature	None	None	–	0

### Histochemical and immunofluorescence staining

Tissue samples were collected for histopathological evaluation through HE staining, as well as for Sirius red and EVG staining. The Sirius red staining procedure (PH1098; Phygene) and EVG staining protocol (Biossci, China) were meticulously executed in accordance with the respective standardized procedures.

For the immunofluorescence analysis, subsequent to the deparaffinization process, antigen retrieval and blocking, the tissue sections underwent exposure to primary antibodies. The primary antibodies utilized were collagen I (1:500, ab270993), collagen III (1:200, ab7778), CD31 (1:500, ab182981), CD86 (1:100, ab238468) and CD206 (1:200, CST #24595). Following the primary antibody exposure, the sections were treated with corresponding secondary antibodies, and nuclear counterstaining was performed using DAPI. Acquisition of fluorescent images was conducted using a Slideview VS200 microscope (Olympus).

### Hydroxyproline content determination

HYP content serves as a vital indicator reflecting collagen tissue metabolism and fibrosis level [43]. The quantification of HYP levels in rat skin tissue was performed utilizing the HYP detection kit (BC0250; Solarbio, Beijing).

### Quantitative real-time polymerase chain reaction

The entirety of SD rat skin tissues was pulverized into powder using liquid nitrogen. RNA extraction was carried out following the protocol of the RNeasy Mini Kit (Qiagen, USA). Subsequent to RNA extraction, reverse transcription into cDNA was executed using the iScript^TM^ cDNA Synthesis Kit (Bio-Rad, USA). Quantitative real-time PCR was performed on the CFX96TM real-time PCR detection system (Bio-Rad, USA) by combining the cDNA solution with SsoFast EvaGreen Supermix (Bio-Rad). The gene expressions of Col I, Col III, elastin and TGF-β were studied. Primer sequences for real-time PCR were detailed in Supplementary Table S1. The gene expression levels of each target gene were determined utilizing the 2^(-ΔΔCt) method, with results normalized to the housekeeping gene glyceraldehyde-3-phosphate dehydrogenase (GAPDH). Triplicates were employed for measurements to calculate means and standard deviations (*n* = 3).

### Statistical analysis

Software GraphPad Prism 7.0 was used for statistical analysis in this study. All data were presented as mean ± standard deviation (SD). To facilitate pairwise comparisons among multiple groups, one- or two-way analysis of variance (ANOVA) with Tukey’s/Šídák’s multiple comparisons test and *t* test was employed. The significance of differences between groups was determined, where *P < 0.05 denoted statistical significance, **P < 0.01 represented a high level of significance and ***P < 0.001 indicated a highly significant disparity.

## Results and discussion

### Observation of changes in photoaging skin

**Figure 1.**
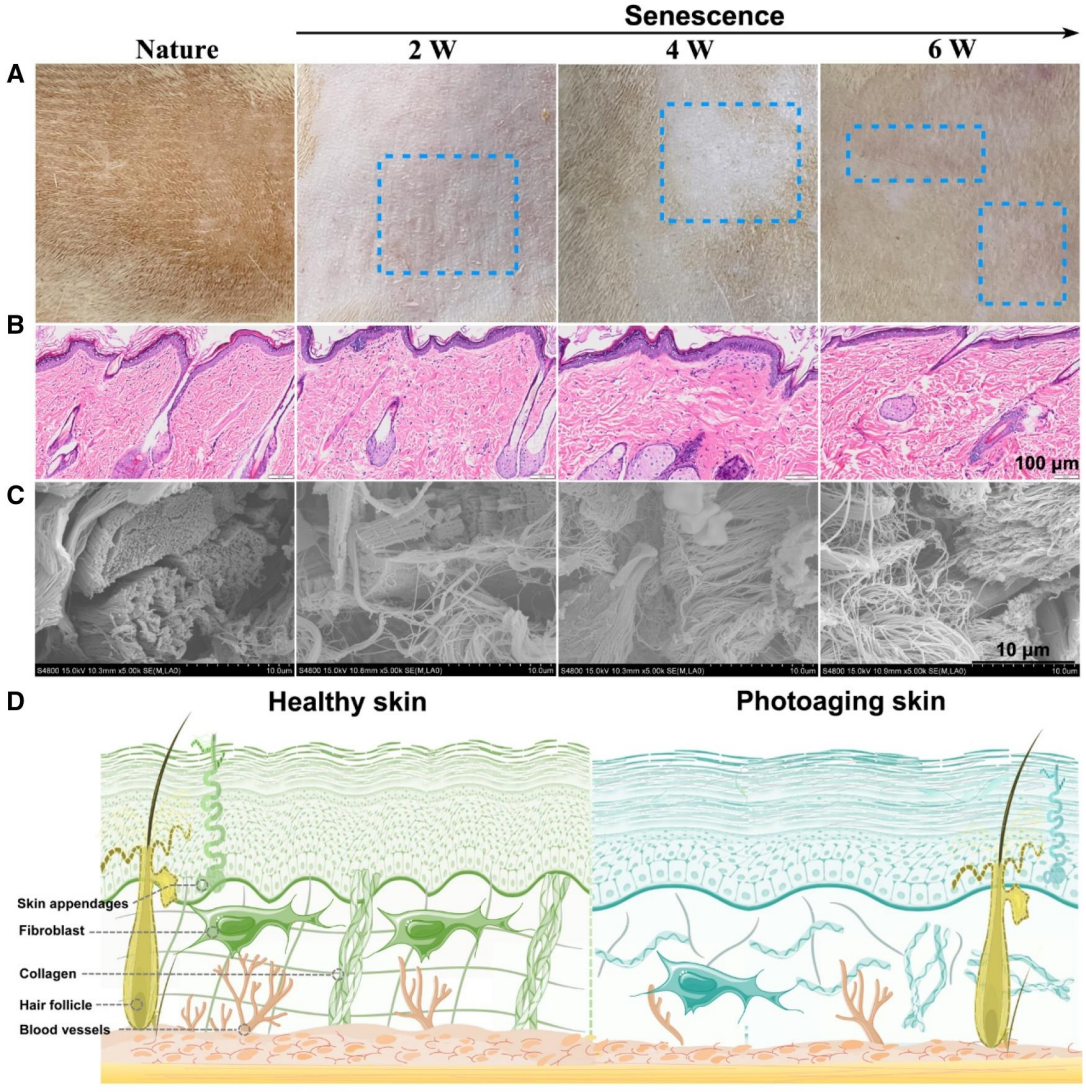
The observation of photoaging skin. (**A**) Macroscopic observation. (**B**) HE staining. (**C**) SEM observation. (**D**) Diagram of changes from healthy skin to photoaging skin.

As can be seen from Figure 1A, the skin showed signs of redness and local whitening after the skin was exposed to ultraviolet radiation for 2, 4 and 6 weeks (blue dotted box). Furthermore, a progressive inhomogeneity in the collagen fibers of the dermis was observed, and distinct gaps between collagen fibers became apparent, transitioning from a natural state to senescence, notably pronounced by the sixth week of photoaging, as evidenced by HE staining (Figure 1B). Subsequent SEM analysis revealed the orderly arrangement of collagen fibers in the natural group. However, in the photoaging group exposed to increasing UV radiation, the dermal collagen fibers exhibited disarray, curling, fracturing, and uneven distribution (Figure 1C and D). In summary, the observed microstructural alterations, encompassing the bending and breaking of collagen fibers, substantiated the great importance of developing bioactive materials to combat skin aging [44].

### Characterization of rhCol III microgel

**Figure 2.**
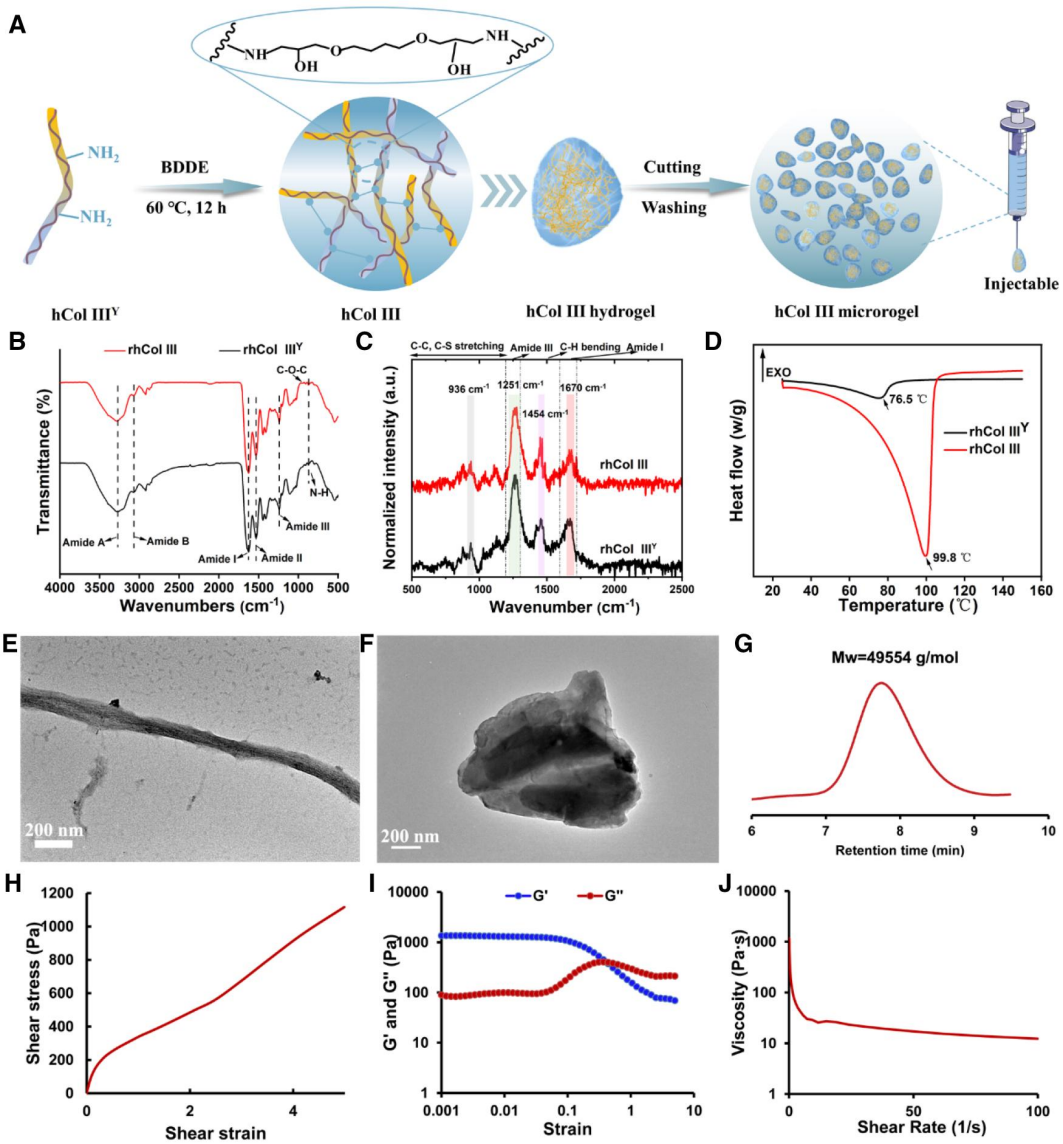
The characterization of rhCol III. (**A**) Schematic diagram of the preparation of injectable rhCol III microgel. (**B**) FTIR characterization of rhCol III^Y^ and rhCol III. (**C**) Raman spectra characterization of rhCol III^Y^ and rhCol III. (**D**) DSC characterization of rhCol III^Y^ and rhCol III. (**E**) The transmission electron microscope (TEM) observation of rhCol III^Y^. (**F**) The TEM observation of rhCol III microgel. (**G**) GPC analysis of rhCol III microgel. (**H**) The diagram of strain–stress for rhCol III microgel. (**I**) The diagram of strain modulus for rhCol III microgel. (**J**) The diagram of shear rate-viscosity for rhCol III microgel (error bars, means ± SD; *n* = 3 per group).

Figure 2A is a schematic diagram of an injectable rhCol III microgel formed from BDDE cross-linked rhCol III^Y^. In the Fourier transform infrared (FTIR) spectrum of rhCol III, the C-O-C was indicated by a peak at 954 cm^−1^, while the characteristic peak of N–H was observed at 869 cm^−1^ (Figure 2B). The results demonstrated the successful cross-linking of rhCol III^Y^ with BDDE. As depicted in Supplementary Table S2, the results showed that the BDDE residual levels in these three batches were determined as “detected, <1 μg/g”, which complies with the product requirement of ≤2 μg/g. As shown in Figure 2B, the characteristic peaks of rhCol III^Y^ and rhCol III showed the same wavenumber, such as amide A (predominantly N–H bending vibrations), amide B (related to CH_2_ stretching vibrations), amide I (the C=O bond stretching vibration), amide II (the N–H bending coupled to C–N stretching) and amide III (the C–N stretching combined with N–H bending) were 3278, 3066, 1623, 1531, 1236 cm^−1^, which were consistent with the collagen absorption peak reported in the literature and also suggested that BDDE cross-linking did not alter the structure of collagen [45]. Furthermore, the secondary structure in detail was investigated using Raman spectroscopy, and the characteristic peak assignments of rhCol III^Y^ and rhCol III in the region 500–2500 cm^−1^ are shown (Figure 2C). The spectra region of 600–1200 cm^−1^ in both spectra was dominated by S–S, C–S and C–C stretching of amino acids such as proline. The wavenumber of amide III and amide I was 1251 and 1670 cm^−1^, which were consistent with findings from FTIR investigations. As shown in Supplementary Figure S1A, rhCol III^Y^ and rhCol III had absorption peaks near 198 nm, indicating a disordered secondary structure [46]. After determining the structure, the thermal stability of rhCol III^Y^ and rhCol III was characterized using thermogravimetric analysis (TG) and DSC. The results of TG exhibited that the mass change of the material had three stages upon heating, containing the rapid decline of 30–100°C, the platform stage of 100–245°C and the rapid decline of 240–640°C, corresponding to the free water dehydration, stabilization and carbonization process of the material (Supplementary Figure S1B). Furthermore, the thermal denaturation temperature of rhCol III^Y^ and rhCol III was 76.5°C, 99.8°C (Figure 2D). The above results suggested that rhCol III had better thermal stability than rhCol III^Y^.

As depicted in Supplementary Figure S1C and D, rhCol III^Y^ exhibited a lamellar structure while rhCol III revealed a porous and loosely particulate distribution. When rhCol III^Y^ was well-distributed in solution, a fibril with a diameter of around 70–120 nm was observed (Figure 2E), which fell within the ranges of collagen fibril diameters reported in the literature (20–500 nm) [47]. The transmission electron microscope results further exhibited that the rhCol III microgel particles showed an irregular shape (Figure 2F). From Supplementary Figure S1E, it was evident that rhCol III microgel in this study exhibited a colorless and transparent appearance. The molecular weight of rhCol III microgel was determined to be 49 554 g/mol through GPC analysis (Figure 2G). To comprehensively evaluate its injectability, we conducted an analysis encompassing osmotic pressure and injectability force. The osmotic pressure was measured at 302.7 ± 1.5 mOsmol/kg (Supplementary Figure S1F), meeting the requirement of osmotic pressure for intradermal injection [48]. The injectability force was determined to be 19.2 ± 0.7 N (Supplementary Figure S1G), marginally exceeding values reported in the literature for hyaluronic acid-based or hyaluronic acid/collagen composite fillers [49, 50]. When microgels are used as fillers for injection, a high level of shear stress needs to be applied. In this regard, the viscoelastic behavior seems to be necessary. Conversely, the injected fillers into facial tissue need elastic behavior to recover the desired volume and shape. In Figure 2H, we found that stress increased with the increasing strain for rhCol III microgel. As exhibited in Figure 2I, within the strain range of 0.01–0.1%, the results of G′>G″ demonstrated that the material exhibited elastic-dominated behavior under static conditions, which might effectively resist external deformation and maintain structural stability at the implantation site. Notably, an intersection point signified the onset of flow within the sample at strains or stress levels beyond this point. Additionally, an increase in shear rate correlated with a reduction in viscosity (Figure 2J), indicative of the shear-thinning behavior of rhCol III microgel. In summary, the rhCol III microgel characterized in this study manifested exceptional injectable properties.

### The interaction of rhCol III microgel with L929 cells

**Figure 3.**
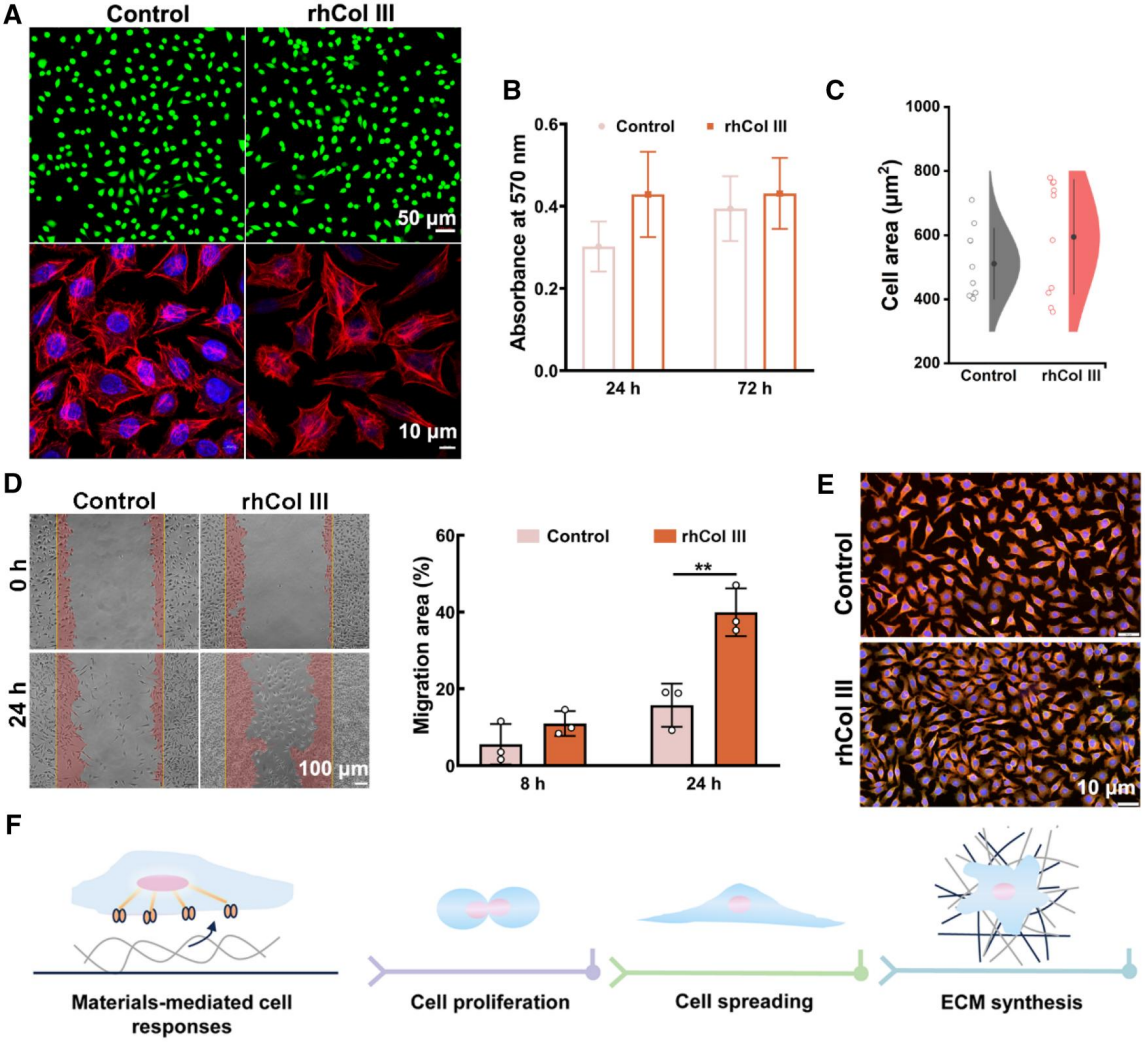
The interaction between cells and rhCol III microgel. (**A**) Representative images illustrating FDA/PI staining and phalloidin/DAPI staining for L929 cells at 3 days in each group. (**B**) Quantification of L929 cells viability. (**C**) The corresponding quantitative assay of the cell area by Phalloidin/DAPI staining. (**D**) Representative images of the migration for L929 cells in each group and quantification of the migration area for L929 cells. (**E**) Immunofluorescent staining for L929 cells subsequent to a 3-day cultivation period. (**F**) Interaction diagram illustrating the relationship between materials and cells. (error bars, means ± SD; *n* = 3 per group; all analyses were done using two-way ANOVA with Šídák’s multiple comparisons test/*t* test). *P < 0.05, **P < 0.01 and ***P < 0.001.

The resultant fluorescence images obtained through live/dead assay (Figure 3A) revealed a widespread and viable distribution of cells on the culture dish, as evidenced by the profusion of green fluorescence dots and almost no red fluorescence dots. This result indicated that rhCol III microgel showed excellent biosafety *in vitro*. Moreover, MTT experiments revealed that OD values increased with longer culture time in all groups, indicating that rhCol III microgel exhibited good biocompatibility and promoted cellular proliferation (Figure 3B). In addition, Figure 3A and C depicts confocal microscopy images of the cytoskeleton-nucleus staining and semi-quantitative analysis on the cytoskeleton staining of the cells at 3 days. It can be observed that cells exposed to rhCol III microgel displayed spindle-shaped cytoskeletal structures similar to Control. Afterward, the migration of L929 cells was investigated (Figure 3D), wherein L929 cells were co-cultured with rhCol III microgel. The results indicated that rhCol III microgel could better promote the cells migration compared to the Control. With the intention of assessing the influence of rhCol III microgel on the secretion of Col I and Col III by L929 cells *in vitro*, co-cultures were established following a 3-day culture period. Immunofluorescent staining for Col I (in green) and Col III (in red) was subsequently conducted. As depicted in Figure 3E, rhCol III and the Control group could promote the collagen secretion by L929 cells. The above results can also be inferred that our material can sustainably promote cell proliferation, migration, morphology maintenance, and collagen secretion (Figure 3F).

### Degradation properties of materials *in vivo*

#### Degradation properties of materials in mice

Combining the analysis of material properties, cell experiments and filling effect, we chose 120 mg/mL rhCol III microgel as the concentration for animal experiments. A subcutaneous injection of 0.2 mL of rhCol III microgel to mice without UV-induced photoaging was performed to investigate the degradation characteristics of rhCol III microgel *in vivo*. Gross examination and HE staining were subsequently conducted. Macroscopic images of the subcutaneous injection of rhCol III microgel in mice were presented in Supplementary Figure S2A, while Supplementary Figure S2B depicts HE staining images obtained from samples collected at 2 and 4 weeks post-subcutaneous injection. It was apparent that the presence of the 0.2 mL rhCol III injected subcutaneously in mice remained noticeable at 2 weeks (red arrow). However, notable degradation of rhCol III was observed at 4 weeks (indicated by the red arrow).

#### Degradation properties of materials in photoaging SD rats

**Figure 4.**
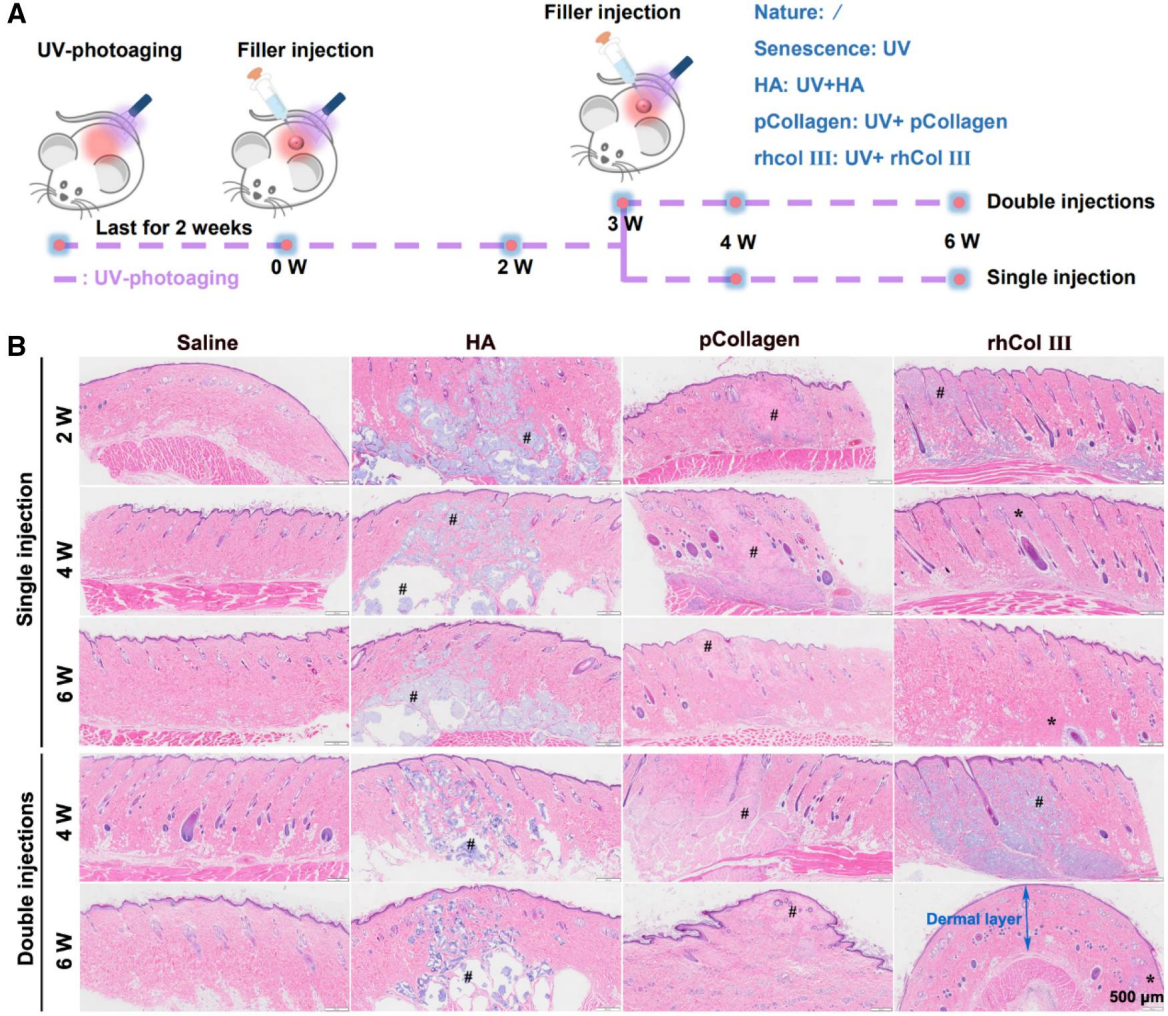
Histological images and corresponding analyses. (**A**) Flow chart delineating the animal experiments. (**B**) The HE staining after materials injection (# denoting the materials; * indicating newly formed tissue).

Figure 4A delineated a schematic diagram of the material injection process *in vivo*. Next, we conducted macroscopic examinations of injection sites over time to enable a comparative analysis of degradation profiles of various injection materials. Supplementary Figure S3 presents macroscopic images capturing the characteristics of different injection materials. The observations derived from Supplementary Figure S3 revealed that the skin of rats injected with physiological saline exhibited no discernible signs of erythema or inflammation, indicating that the injection procedure did not induce adverse reactions. However, upon injecting the materials into the dermal layer, the absence of skin redness around the injection sites for all three materials was noted. This observation suggested that the utilization of the filler materials may contribute to an improvement in the transition of skin to a healthier state. In the context of injection volumes of 0.5 mL distributed across ten injection points, it was observed that rhCol III microgel was no longer visibly present at the injection sites in both the single injection group and the double injection group by the sixth week. In contrast, materials from HA and pCollagen remained detectable at the sixth week in both the single injection group and the double injections group (black arrow). This suggested that the degradation rate of rhCol III microgel might be relatively faster than that of HA and pCollagen.

As illustrated in Figure 4B, HE staining revealed that rhCol III microgel was no longer evident at 4 and 6 weeks in the single injection group (* denoted newly formed tissue at the injection site). However, in the double injections group, the rhCol III microgel was still visibly present (# represented material) at 4 weeks, becoming nearly imperceptible at 6 weeks. For filling HA and pCollagen, material presence was observable from the second week to the sixth week after material injection (# represented material), both in the single injection group and the double injections group (Figure 4B). Nevertheless, voids were evident in the injection sites of the HA group, aligning with findings in existing literature [51]. Therefore, the aforementioned observations indicated that the degradation rate of rhCol III microgel might be relatively faster than that of HA and pCollagen under the same injection volume, confirming alignment with macroscopic examination results.

The degradation performance of dermal filler materials is a critical factor influencing their efficacy [52]. Therefore, in the development of filler materials, it is essential to align the degradation rate with the regeneration rate of new collagen [53]. Upon analyzing the materials in this study, it becomes evident that HA predominantly consists of modified sodium hyaluronate gel. However, as observed in Figure 4B, despite its relatively slow degradation, the presence of voids around the injection site of HA was noticeable. pCollagen is primarily composed of cross-linked Col I purified from pig skin, featuring a unique triple helix structure with approximately 3000 amino acids and a molecular weight of around 300 000 g/mol [54, 55]. In comparison, rhCol III expressed in Pichia pastoris yeast has a close resemblance of amino acid composition to native human collagen, with a molecular weight of 49 554 g/mol. The protein structure of rhCol III consists entirely of Gly-X-Y triple peptide repeat sequences without the triple helix structure. Consequently, its degradation rate was faster than that of pCollagen mainly due to the distinct collagen structure. Some literature reported that rhCol III exhibited cell adhesion performance superior to collagen derived from animals [56]. Hence, the evaluation of the efficacy of filler materials necessitated consideration not only of their safety and degradation characteristics *in vivo* but also of factors such as raw material quality and the effectiveness in inducing collagen regeneration within the body.

### Evaluation of biosafety of rhCol III *in vivo*

**Figure 5.**
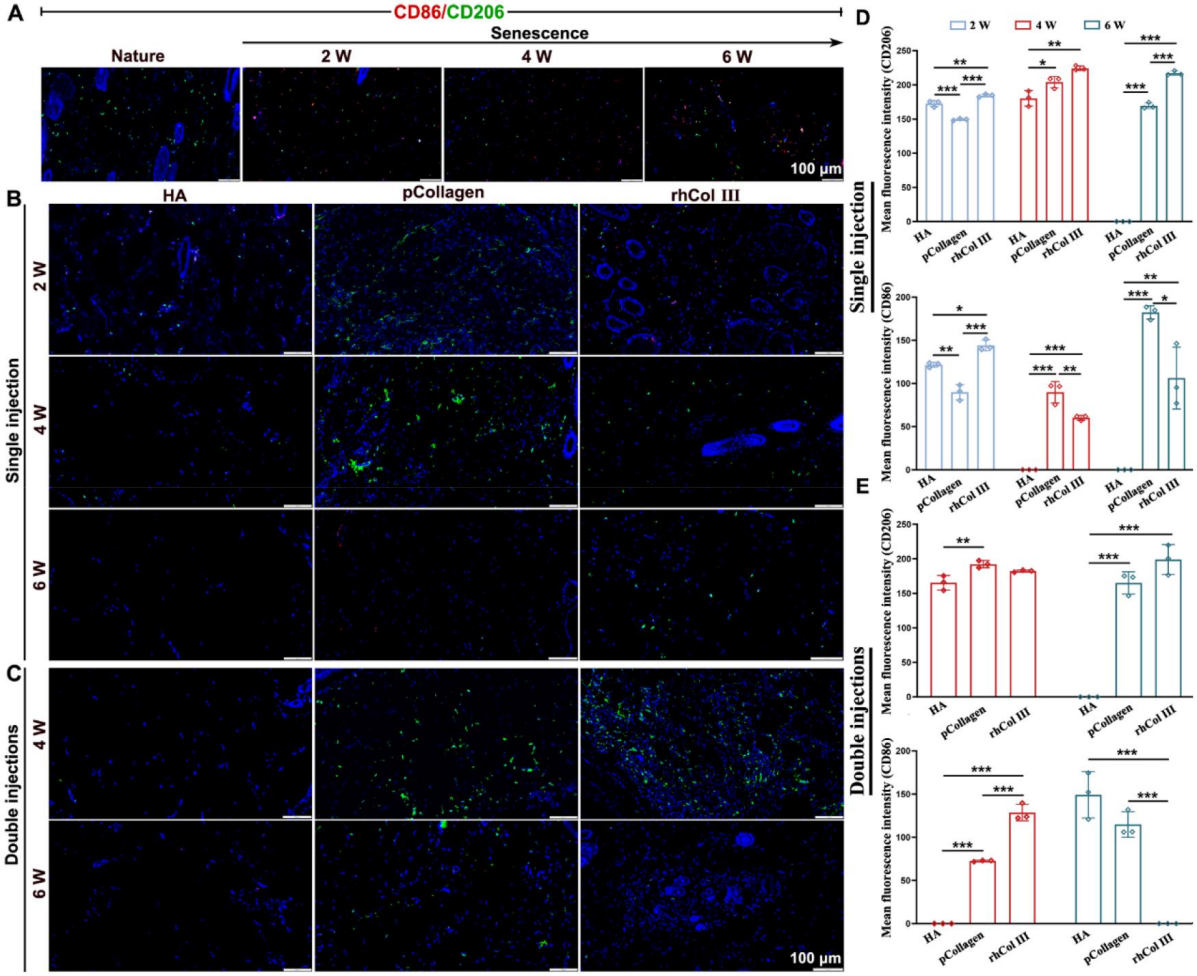
Biosafety evaluation of materials injected into the dermis. (**A**) The CD86/CD206 immunofluorescence staining for both natural and senescent samples. (**B** and **C**) The CD86/CD206 immunofluorescence staining at the injection sites of materials. (**D** and **E**) The mean fluorescence intensity of CD86 and CD206.

The assessment of macrophage phenotypes recruited to the injury site serves as a crucial indicator of the inflammatory response. M1-type macrophages are known to promote inflammation, whereas M2-type macrophages play a role in inhibiting inflammation and fostering tissue regeneration [57]. In order to provide a comprehensive illustration of the inflammation levels at the wound sites, the infiltration of macrophages was examined through immunofluorescence staining, with CD86 and CD206 chosen as representative surface markers for M1 or M2 macrophages, respectively. As depicted in Figure 5A, the senescence group, which did not undergo material injection, exhibited an increased expression of CD86, while CD206-positive macrophages decreased as skin senescence progressed from 2 to 6 weeks, indicating heightened inflammation during the progression of photoaging. However, upon injection of material into the dermal layer at 2 weeks, there was a decrease in the proportion of M1 macrophages, indicating a mitigated inflammatory state (Figure 5B–E). However, with prolonged time, it became evident that the proportion of M2 macrophages surpassed that of M1 macrophages in the rhCol III microgel after 2 weeks of injection (Figure 5B–E). In summary, the rhCol III microgel injection proved effective in ameliorating the local inflammatory response and transforming the tissue microenvironment from an inflammatory to a reparative state. This might be attributed to the polarization of macrophages to the M2 type, which in turn promoted the secretion of anti-inflammatory factors in aging skin, irrespective of the injection frequency (single or double injections).

### The impact of material injection on elastic fiber

**Figure 6.**
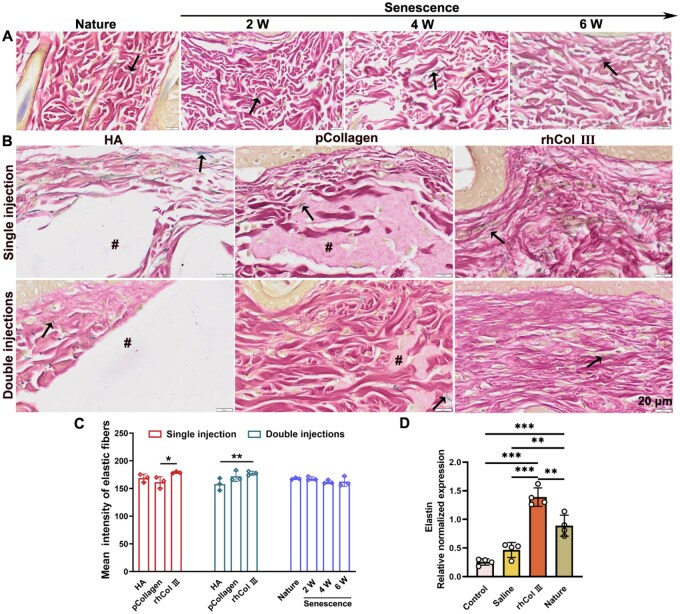
Exploring the impact of materials injection on elastic fibers. (**A**) EVG staining of both natural and senescent groups (black arrow representing elastic fibers). (**B**) EVG staining after materials injection (# signifying materials). (**C**) The mean intensity of elastic fibers. (**D**) Evaluation of relative gene expression levels (error bars, means ± SD; *n* = 4 per group; all analyses were done using one-way ANOVA with Tukey’s multiple comparisons test). *P < 0.05, **P < 0.01 and ***P < 0.001.

Skin aging is a multifaceted biological process primarily impacting the dermis [29, 44]. Elastic fibers, integral to maintaining skin elasticity by forming a network in the dermis, constitute 2–4% of the skin’s ECM, playing a crucial role in the aging process [58]. To evaluate the potential impact of material injections on dermal elastic fibers, EVG staining was performed at injection sites, with collagen fibers stained in red and elastic fibers in black. In the natural group, elastic fibers appeared as slender black strands interwoven between collagen fibers (Figure 6A). However, as skin aged over 2–6 weeks, collagen fibers in the dermal layer exhibited fragmentation and decreased in quantity (Figure 6A–C). Following HA injection, disruption to both collagen and elastic fibers, resulting in voids, was observed (Figure 6B and Supplementary Figure S4). In contrast, pCollagen injection caused minimal disruption to dermal collagen and elastic fibers, while rhCol III microgel injection distributed the material between collagen and elastic fibers without voids (Figure 6B and Supplementary Figure S4). At 6 weeks, a noteworthy difference in the rhCol III microgel group compared to the HA group was the observation of elastic fibers (black arrow) in the materials injection site after rhCol III microgel degradation. Additionally, dark elastic fibers were present in newly generated collagen in the rhCol III microgel group, while the pCollagen group exhibited black fibers located between the material (#) and the pre-existing tissue. Conversely, a double injection of rhCol III microgel led to the regeneration of collagen and elastic fibers more closely resembling those in the natural group. As shown in Figure 6C, the mean intensity of elastic fibers was higher in the rhCol III group compared to both HA and pCollagen under a single injection or double injections. Compared to the senescence and saline cohorts, the rhCol III microgel group demonstrated a significant upregulation in the expression of elastin, even beyond the natural group (Figure 6D). In summary, these findings suggested that the introduction of rhCol III microgel might hold promise for stimulating elastin regeneration *in vivo*.

### The ability of the material to induce the regeneration of collagen *in vivo*

Angiogenesis is a vital process essential for tissue remodeling [59]. CD31, a transmembrane protein expressed during early angiogenesis, serves as a marker for neovascularization [60]. As illustrated in Supplementary Figure S5, a notable enhancement in angiogenesis was observed in both the pCollagen and rhCol III microgel groups at 6 weeks, contrasting with the HA and natural groups. Collectively, the pCollagen and rhCol III microgel could effectively enhance angiogenesis, thereby facilitating the accelerated regeneration of newly formed collagen.

**Figure 7.**
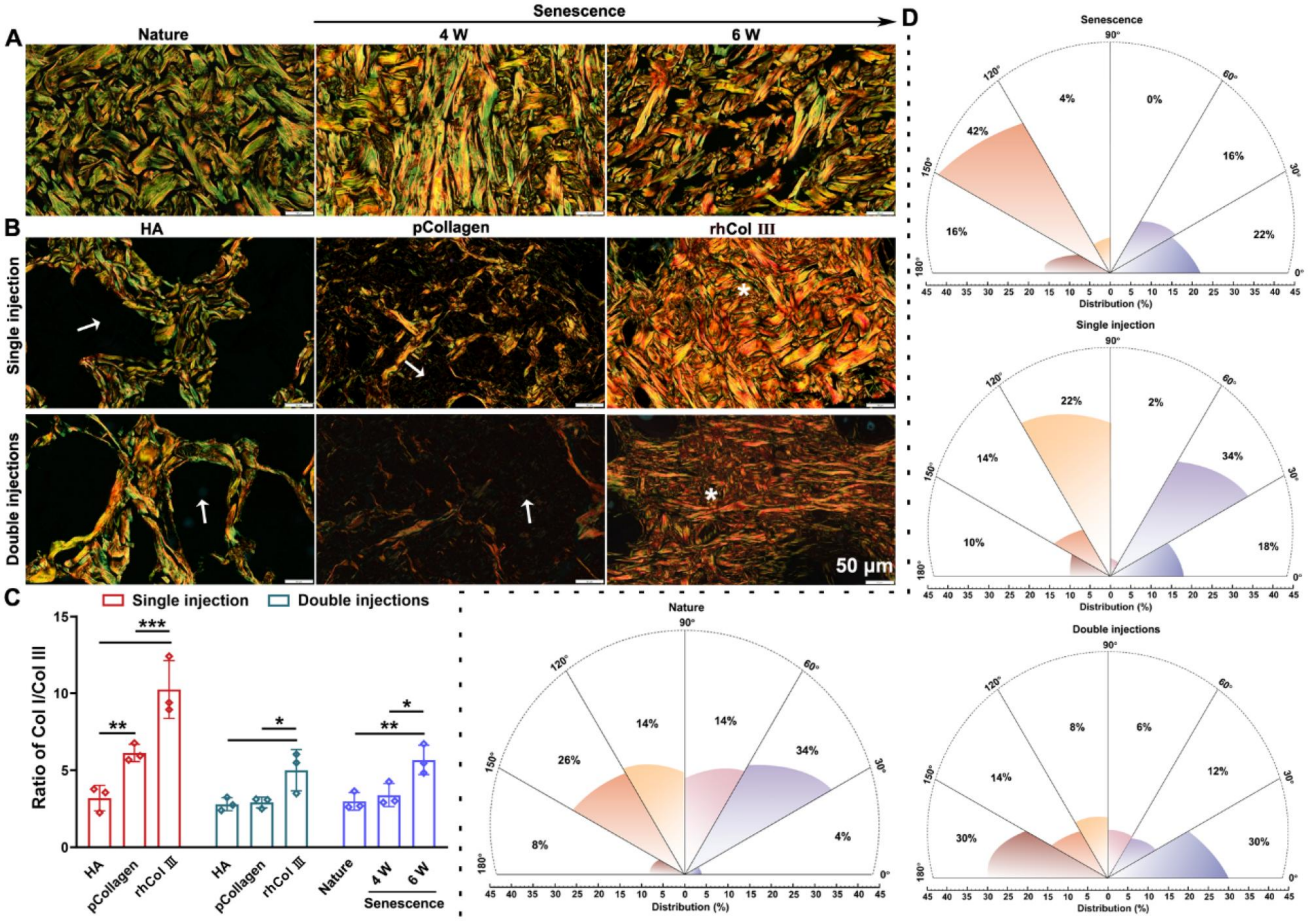
Exploring the impact of materials injection on collagen fibers. (**A**) Sirius red staining of both natural and senescent samples at high magnification. (**B**) Sirius red staining at materials injection sites at high magnification (white arrow indicating materials; * signifying newly formed collagen fibers). (**C**) The ratio of Col I/Col III. (**D**) Analysis of collagen fiber orientation following rhCol III microgel injection at 6 weeks. (*n* = 3 per group).

Collagen fibers maintain the skin at a certain tension and toughness [13]. To further investigate the induction of collagen regeneration in the dermal layer subsequent to material injection, we employed Sirius red staining coupled with relevant biological quantification. Col I fibers, characterized by tight arrangement and strong birefringence, manifested as yellow or red fibers, while Col III fibers, exhibiting weaker birefringence, presented as fine green fibers. Figure 7A and B showcased Sirius red staining images following material injection. In Figure 7A, the collagen fibers in the natural group displayed a mesh-like woven structure. As the senescence process advanced from 4 to 6 weeks, there was a gradual disarray in the organization of collagen fibers, accompanied by a change in their orientation. By the sixth week, conspicuous gaps between collagen fibers became evident. As illustrated in Figure 7B, the HA and pCollagen group exhibited voids between collagen fibers. In contrast, following the degradation of rhCol III microgel in both the single and the double injections groups at 6 weeks, no voids were observed, and compact collagen was evident in the rhCol III microgel group. As can be seen from Figure 7C, the ratio of Col I/Col III gradually increased from nature to 6 weeks of aging, indicating a gradual decrease in Col III relative to Col I as the skin aged. Regardless of single or double injections, the rhCol III group showed a higher Col I/Col III ratio than the HA group, the pCollagen group and the nature group. These findings indicated that the injection of rhCol III might induce the secretion of Col I.

Additionally, an analysis of collagen fiber orientation (Figure 7D) indicated that in the natural collagen, it was predominantly distributed between 30° and 60°, constituting 34%. Conversely, in the senescence group, collagen fiber orientation was most prevalent between 120° and 150°, comprising 42%. After dermal injection of rhCol III microgel for 6 weeks, single injection of rhCol III microgel exhibited a distribution primarily between 30° and 60°, constituting 34%, while double injections of rhCol III microgel displayed a distribution mainly between 0–30° and 150–180°, both at 30%. These results suggested that the orientation of newly formed collagen fibers after a single injection of rhCol III microgel closely approximated that of the natural group, indicating superior effects compared to double injections of rhCol III microgel. These findings underscored the potential of material injection in delaying or reversing the senescent state of the skin.

**Figure 8.**
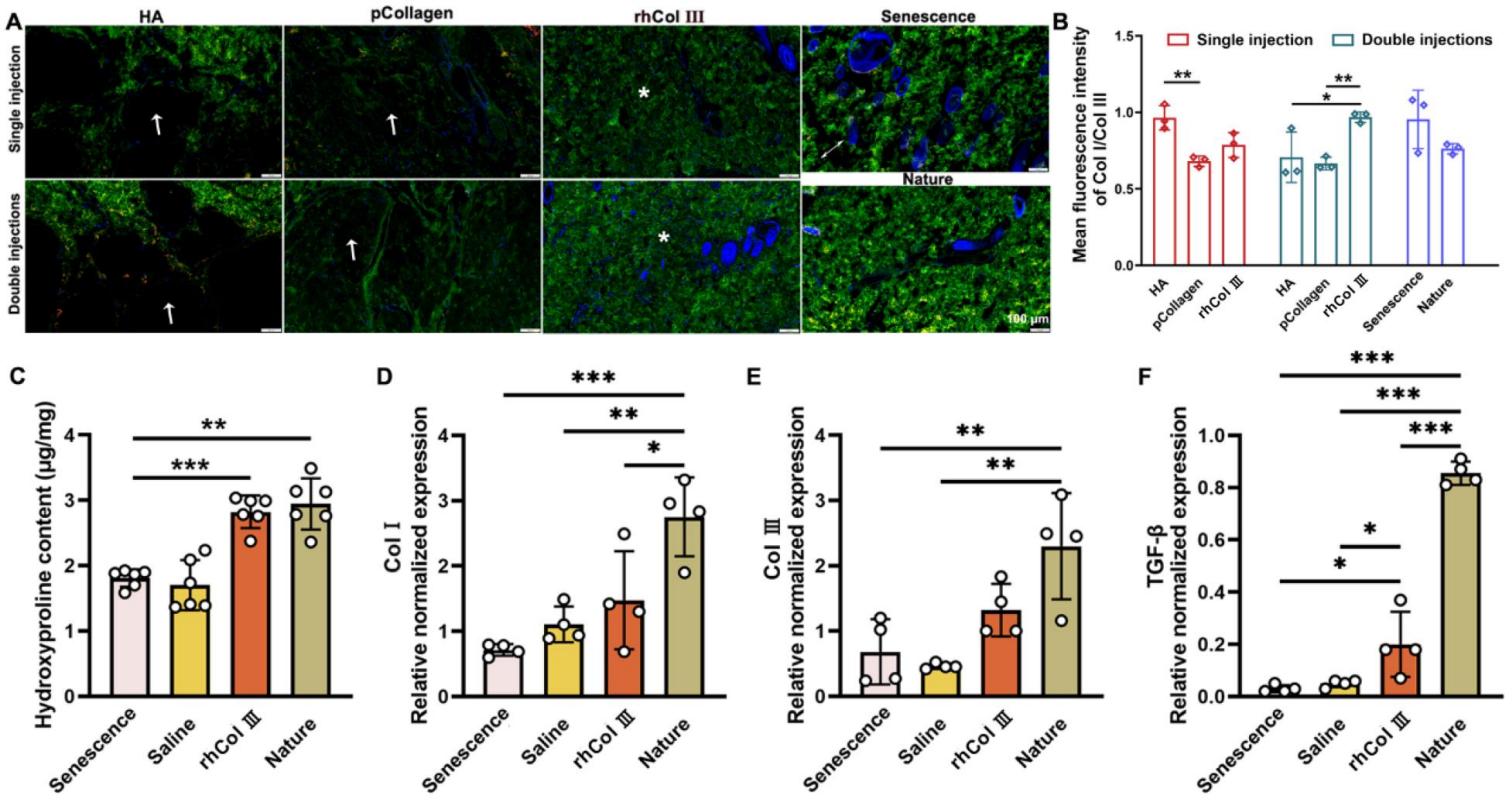
Evaluation of collagen regeneration *in vivo*. (**A**) Immunofluorescence staining for Col I and Col III at materials injection sites at 6 weeks (white arrow denoting materials; * signifying newly synthesized collagen fibers). (*n* = 3 per group). (**B**) The mean fluorescence intensity of Col I/Col III. (**C**) Quantitative assessment of hydroxyproline content from materials injection sites in double injections groups at 6 weeks. (**D–F**) Evaluation of relative gene expression levels from materials injection sites in double injections groups at 6 weeks (error bars, means ± SD; *n* = 4 per group; all analyses were done using one-way ANOVA with Tukey’s multiple comparisons test). *P < 0.05, **P < 0.01 and ***P < 0.001.

To more accurately evaluate alterations in newly synthesized collagen at the injection site, we conducted immunofluorescent staining (Col I (green) and Col III (red)). As shown in Figure 8A and B, the dermal layer exhibited substantial expression of both Col I and Col III in natural samples. However, after 6 weeks of senescence, noticeable gaps appeared between collagen fibers. As depicted in Figure 8A and B, the secretion of Col I/Col III was most prominent in the vicinity of material injection at 6 weeks with a single injection in the HA group, but voids accompanied these observations. The pCollagen group consistently exhibited Col I secretion near the material injection site, with only a small amount of Col I/Col III expression at 6 weeks in the single injection group and double injections group (Figure 8A and B). With the degradation of the rhCol III microgel, compared to the pCollagen group and the HA group, the rhCol III microgel group produced more Col I/Col III at 6 weeks in the double injections group. However, there was no significant difference observed between single injection and double injections of rhCol III microgel. These results indicated that rhCol III microgel might effectively induce the production of new Col I and Col III, making it an ideal filling material.

Quantification of hydroxyproline (Figure 8C) and the corresponding gene expression analysis (Figure 8D–F) were conducted to assess the impact of various materials injected into the rat dermis. The hydroxyproline analysis revealed that the rhCol III microgel group exhibited hydroxyproline levels proximal to those observed in the natural group, surpassing other groups. Comparative to the senescence and saline cohorts, the rhCol III microgel group demonstrated the highest expression of TGF-β, Col III, and Col I, aligning more closely with the natural group. These findings suggested that the introduction of rhCol III microgel might hold promise for stimulating collagen regeneration *in vivo*.

Collagen depletion stands as a pivotal factor in the aging process of the skin, given its synthesis and secretion by fibroblasts. Age-related alterations manifest as a progressive decline in Col III, accompanied by breakage of some Col I [21]. Consequently, skin undergoes atrophy, reduced thickness, and the emergence of wrinkles, indicative of the aging phenomenon [61]. To address this concern, the current market primarily offers fillers, with HA being a choice. However, HA fillers have limitations, including the product migration, late swelling, and inflammatory reactions [62]. Alternatively, collagen-based fillers, with pCollagen as a notable representative, present heightened efficacy. In terms of filling efficacy, collagen injections yield more natural outcomes and exhibit reduced susceptibility to deformation arising from water absorption and migration [63]. Based on our experimental findings, rhCol III microgel elicited the generation of novel elastic fibers and collagen fibers in comparison to pCollagen (Figures 6–8). The alignment and orientation of collagen fibers closely approximated those observed in natural skin specimens. This phenomenon was ascribed to the genetic engineering of the recombinant human collagen employed in this investigation, which was designed based on the amino acid sequence of human type III collagen. The genetic construct was specifically devised to enhance collagen solubility, elevate expression levels, and concurrently preserve the distinctive attributes of collagen. The resultant protein comprises exclusively Gly-X-Y tripeptide repeat sequences, mirroring the amino acid composition akin to natural human collagen [31]. Nevertheless, alterations have been made to the sequence arrangement, wherein hydrophobic amino acids have been substituted with hydrophilic amino acids to augment the hydrophilicity of the recombinant human collagen [64]. Consequently, it exhibits a distinctive chemical structure and superior performance in contrast to collagen derived from animal sources. Literature revealed that intradermal administration of rhCol III leads to a notable downregulation in the gene expression of matrix metalloproteinase 3 and an increase in superoxide dismutase. These molecular events culminated in a consequential augmentation of the expression levels of Col I and Col III [29]. Some researchers previously reported that rhCol III possessed abundant integrin recognition sites, which could interact with receptors on the fibroblast surface to modulate cellular behavior, remodeling of the ECM. Also, the recognition sites were beneficial for the remodeling of the collagen network and further enhanced the mechanical performance of the damaged ECM, such as the tensile strength of the tissue (skin elasticity) [29].

Various dermal fillers exhibit distinct characteristics, associated risks, and injection requirements. The potential complications are inherent in all dermal fillers, primarily contingent upon dosage and technique, though some may be linked to the material composition itself [65]. Nevertheless, under the condition of ensuring favorable outcomes, a decrease in the injection frequency holds the potential to partially alleviate the incidence of adverse events. The mutual goal of physicians and patients alike is to achieve superior results with minimal dosage and application frequency. Within the scope of this study, a comparative analysis was conducted between a single injection and two injections of HA, pCollagen, and rhCol III microgel. The findings indicated that, upon visual assessment 4 weeks post-injection, the presence of injection shape was notably more prominent following double injections compared to single injection for HA, pCollagen and rhCol III microgel (Supplementary Figure S3). However, the degradation rate and neo-collagen regeneration were not evident for single and double injections during the 4–6 weeks post-injection of HA and pCollagen (Figures 4, 7, and 8). HE staining discerned that both single and double injections of rhCol III microgel exhibited degradation, thereby fostering new tissue formation by the sixth week (Figure 4). Both single and double injections of rhCol III microgel demonstrated the capacity to engender newly formed collagen and elastic fibers, with marginal distinctions in the quantity of newly generated collagen. However, subsequent to a single injection of rhCol III microgel, the configuration of the newly formed collagen and the quantity of elastic fibers more closely approximated those of the natural group compared to double injections (Figures 6B, 7B, and 8). One reason might be attributed to the fact that the entry of material during the second injection destroyed the collagen and elastic fibers formed by the first injection. The other reason was that it took time for forming mature collagen and elastic fiber. Consequently, the rhCol III microgel employed herein exhibited promise in eliciting sustained collagen production and reinstating collagen architecture without necessitating repetitive injections, thereby highlighting promising prospects for pragmatic application.

Although this study confirmed the effect of rhCol III microgel dermal injection on promoting collagen regeneration, further assessment with specific instruments and biochemical analysis is required to evaluate its anti-aging effects on the skin accurately.

## Conclusion

RhCol III emerges as a promising substrate for surgical implants and scaffolds within the domain of tissue-engineered medical products, circumventing the drawbacks associated with animal-derived collagen. In our study, the rhCol III microgel exhibited a disordered secondary structure and indicated excellent stability. Furthermore, the rhCol III microgel demonstrated superior biocompatibility, promoting the proliferation and migration of L929 cells. Notably, the implantation of rhCol III microgel in skin photoaging animal model demonstrated superior efficacy in reconstructing the ECM homeostasis by fostering the synthesis of collagen and elastic fibers, surpassing other fillers. Our experimental outcomes further unveiled that, when compared to the double injections of rhCol III microgel, single injection of rhCol III microgel yielded collagen structures more closely resembling those found in the natural group, thereby effectively rejuvenating aging skin. The specific mechanism and applicability need more in-depth and extensive research in the future. As medical cosmetology becomes more common, rhCol III microgel shows considerable promise and may become popular for anti-aging in the future, as well as for other ways of improving skin problems.

## Supplementary Material

rbaf076_Supplementary_Data
