# Supplementary material for: Recombinant human collagen type III microgel: an advanced injectable dermal filler for rejuvenating aging skin
Source: Regen Biomater. 2025 Jul 28;12:rbaf076. doi: 10.1093/rb/rbaf076 (PMC12368855; doi:10.1093/rb/rbaf076)
Supplement: rbaf076_Supplementary_Data [file rbaf076_supplementary_data.docx]

**Recombinant human collagen type III microgel: an advanced injectable dermal filler for rejuvenating aging skin**

Yafang Chen^a,b^, Yihan Zhao^a,b^, Xinyue Zhang^a,^^b^, Yang Sun^a,b,f^, Kang Li^a,b,d^, Liguo Zhang^e^, Shuang Li^e^, Jie Liang^a,b,c^, Kefeng Wang ^a,b*^, Yujiang Fan^a,b^^*^

^a^National Engineering Research Center for Biomaterials, Sichuan University, 29# Wangjiang Road, Chengdu 610065, China.

^b^College of Biomedical Engineering, Sichuan University, 29# Wangjiang Road, Chengdu 610065, China.

^c^Sichuan Testing Center for Biomaterials and Medical Devices, Chengdu 610064, China.

^d^State Key Laboratory for Conservation and Utilization of Subtropical Agro-bioresources, College of Life Science and Technology, Guangxi University, Nanning 530004, China.

^e^Harbin Fuerjia Technology Co., Ltd. Harbin 150000, China.

^f^Collaborative Innovation Centre of Regenerative Medicine and Medical BioResource Development and Application Co-constructed by the Province and Ministry, Guangxi Medical University, Nanning, Guangxi 530021, China.

*Corresponding address.

Email: fencal@scu.edu.cn, [fan_yujiang@scu.edu.cn](mailto:fan_yujiang@scu.edu.cn)

**Table S1.** The primer sequences of the target gene for RT-qPCR.

| Species | Gene name | Direction | | Sequence (5'-3') |
| --- | --- | --- | --- | --- |
| Mouse | IL-1β | | Forward | TGCCACCTTTTGACAGTGATG |
|  |  |  | Reverse | CCTGAAGCTCTTGTTGATGTGC |
| Mouse | GAPDH | | Forward | TCGGAGTGAACGGATTTGGC |
|  |  |  | Reverse | TTCCCGTTCTCAGCCTTGAC |
| Rat | Col Ⅰ | | Forward | GAGAACCAGCAGAGCCA |
|  |  |  | Reverse | GAACAAGGTGACAGAGGCATA |
| Rat | Col Ⅲ | | Forward | GTGCTACTGTGAGCTGCTTCTTC |
|  |  |  | Reverse | TCTACATTGGACTGCTGTGCC |
| Rat | Elastin | | Forward | GGTGATCTTGGAGGAGCAGG |
|  |  |  | Reverse | CCAGCTCCAAATCCAGGGAC |
| Rat | TGF-β | | Forward | GTCAGCTGGCCTCGGTC |
|  |  |  | Reverse | ATGACAGTGCGGTTATGGCA |
| Rat | GAPDH | | Forward | AGACAGCCGCATCTTCTTGT |
|  |  |  | Reverse | TTCCCATTCTCAGCCTTGAC |

**Table S2.** The residual amount of BDDE in the sample.

| **Sample** | **Peak area**  **（Ai, pA.s）** | | **Sample weight（Wi, g）** | **Detection limit（μg/g）** | **Residual amount of BDDE（μg/g）** | **Limiting value（μg/g）** |
| --- | --- | --- | --- | --- | --- | --- |
| 1 | | Not detected | 1.0055 | 1 | / | 2 |
| 2 | | Not detected | 1.0061 |  | / |  |
| 3 | | Not detected | 1.0034 |  | / |  |


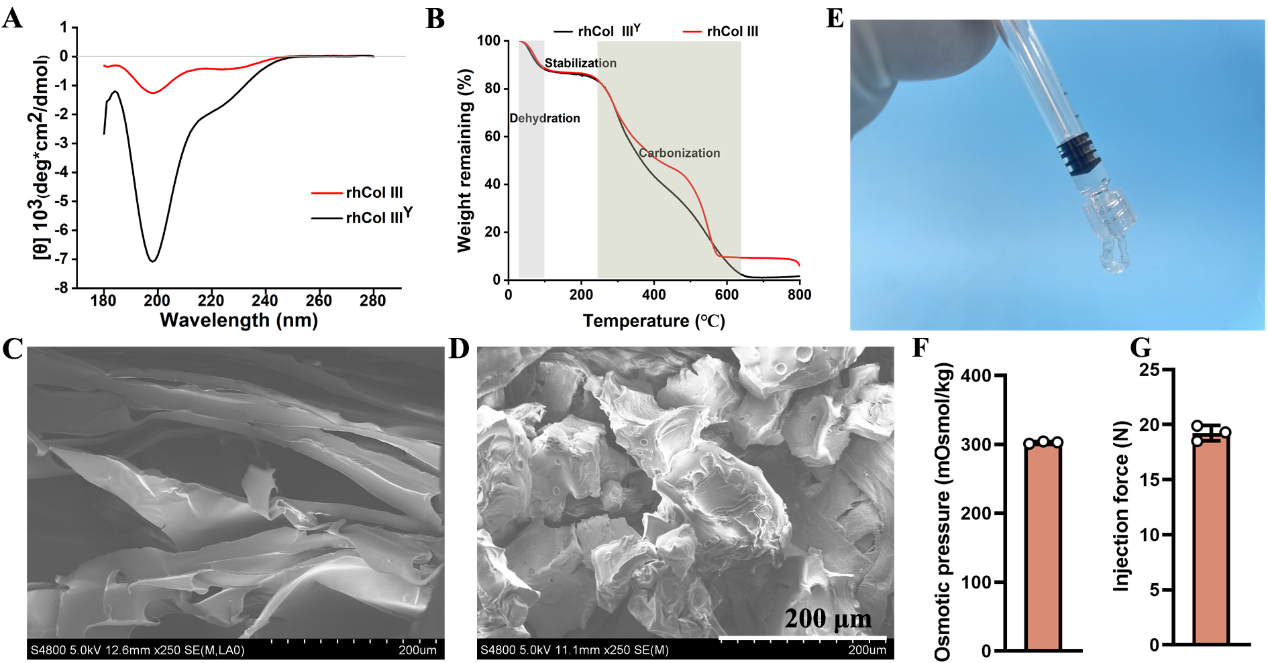


**Figure S1.** The characterization of rhCol III^Y^ and rhCol III. (**A**) The CD spectra of characterization of rhCol III^Y^ and rhCol III at room temperature. (**B**) TG characterization of rhCol III^Y^ and rhCol III. (**C**) The SEM observation of rhCol III^Y^. (**D**) The SEM observation of rhCol III. (**E**) Macroscopical picture of rhCol III injection. (**F**) The osmotic pressure of rhCol III. (**G**) The injection force of rhCol III.


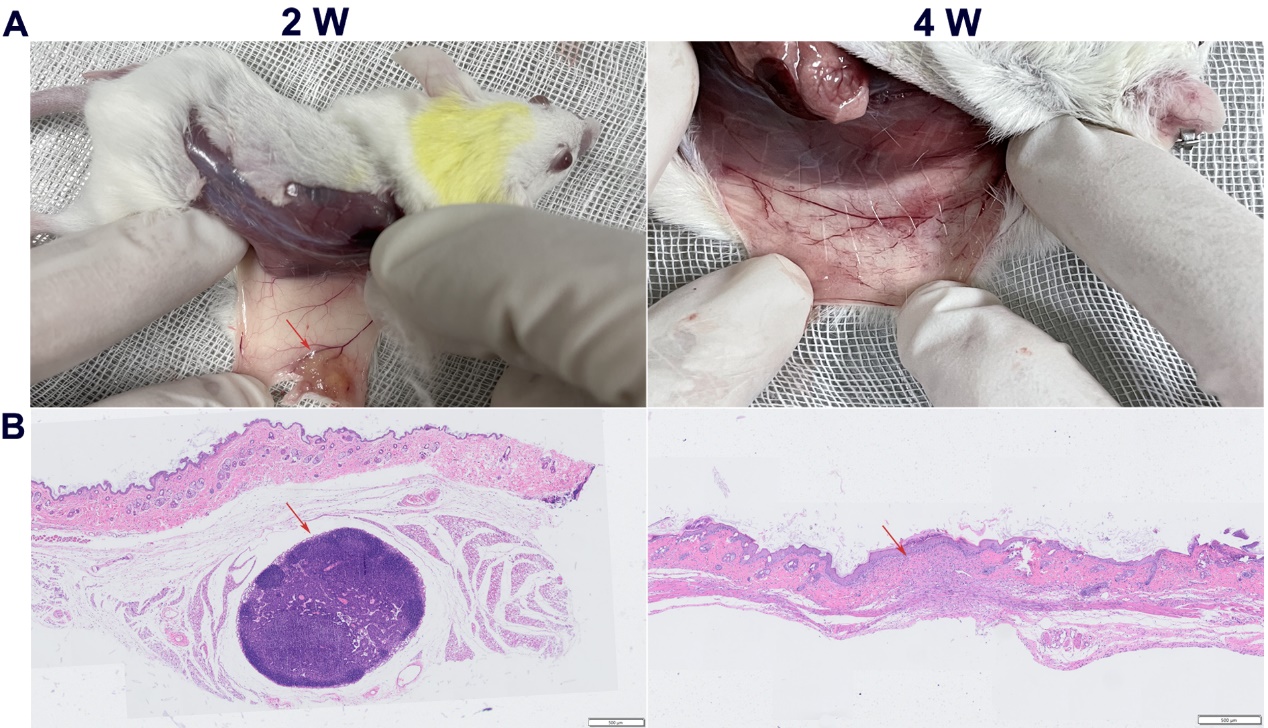


**Figure S2.** Assessment of degradation properties following materials injection in normal mice. (**A**) Macroscopic examination post-materials injection at 2 weeks and 4 weeks. (**B**) HE staining subsequent to materials injection at 2 weeks and 4 weeks (red arrow denoting the materials).


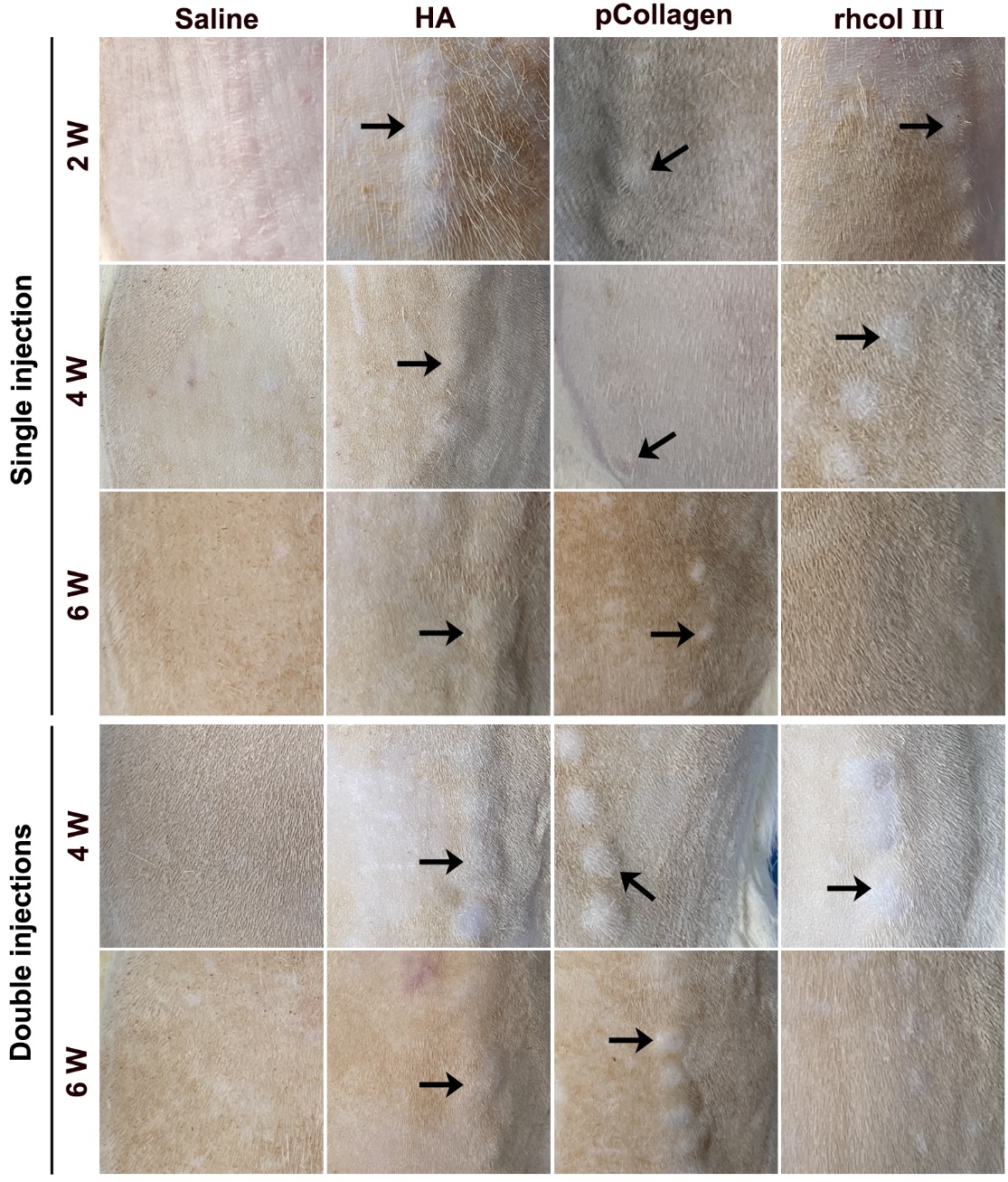


**Figure S3.** The comparation of degradation characteristics following different materials injections under skin photoaging conditions in SD rats (black arrow indicating materials injection sites).


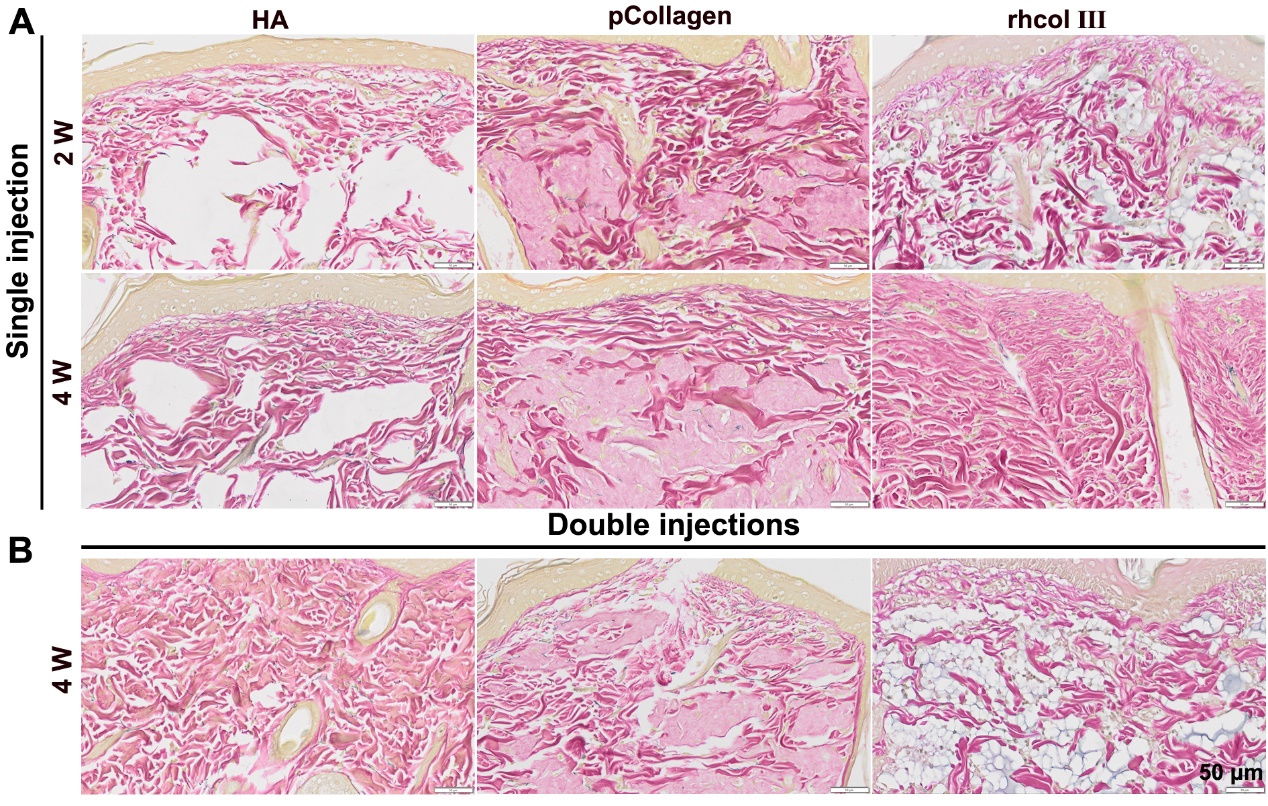


**Figure S4.** Investigating the impact of materials injection on elastic fibers. (**A**) EVG staining in single injection groups. (**B**) EVG staining in double injections groups.

**
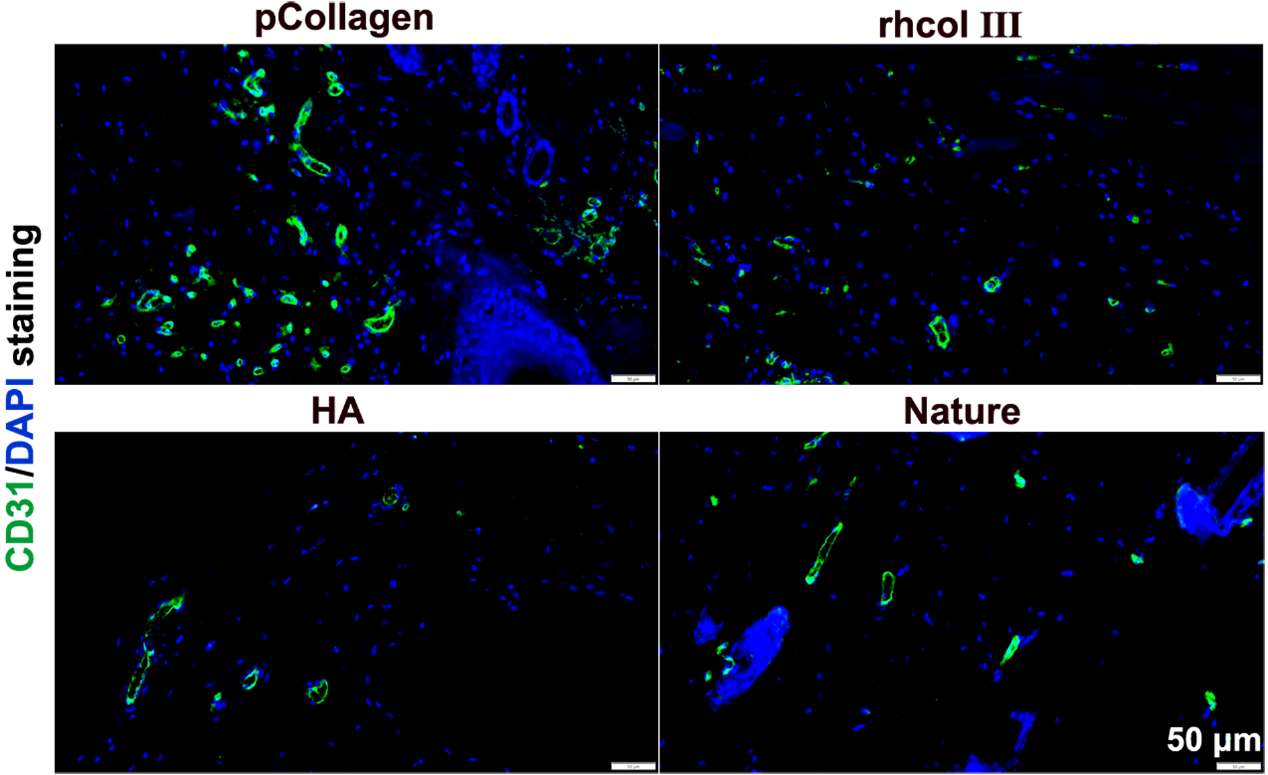
**

**Figure S5.** Assessment of angiogenic properties at injection sites in single injection groups through CD31 immunofluorescence staining at 6 weeks.
